# Enhancing intravesical bladder cancer therapy: from mucoadhesive to targeted drug delivery strategies

**DOI:** 10.3389/fmed.2026.1979620

**Published:** 2026-09-28

**Authors:** Hatixhe Sahini, Maria Anzengruber, Ulrich Lächelt

**Affiliations:** 1 Department of Pharmaceutical Sciences, University of Vienna, Vienna, Austria; 2 Vienna Doctoral School of Pharmaceutical, Nutritional and Sport Sciences, University of Vienna, Vienna, Austria

**Keywords:** bladder cancer, drug delivery, intravesical therapy, mucoadhesion, receptor targeting

## Abstract

Intravesical administration is a standard application route for the treatment of non-muscle-invasive bladder cancer, as it allows for high drug concentrations near tumor sites while largely avoiding systemic exposure and associated adverse effects. However, the efficacy can be constrained by the urothelium's strong barrier properties, as well as continuous dilution and elimination of drugs through urine production and voiding. Bladder-retentive drug delivery systems, such as intravesical sustained-release formulations that accumulate therapeutics at the bladder wall or active targeting to tumor cells may address these limitations. In this context, mucoadhesive formulations are promising because they resist urinary washout, extend dwell time, and enhance urothelial drug deposition. Targeted delivery platforms can increase drug load within malignant tissues and may even accelerate onset of pharmacologic activity via receptor mediated pathways. If binding to tumor-specific target structures also modulates the disease itself, active targeting can extend beyond pharmacokinetic benefits to integrate different pharmacodynamic mechanisms, thereby contributing to more precise and effective cancer therapy. Several targeted intravesical drug formulations, including antibody-drug conjugates against tumor associated antigens, integrin directed systems, hyaluronic acid–based CD44 targeted constructs, as well as immunomodulatory agents, have advanced to clinical or late preclinical evaluation. The intravesical route further provides an opportunity to repurpose drugs originally developed for systemic use but limited by off target toxicities that are unlikely to be dose limiting with local intravesical administration. This mini review provides an overview of intravesical, bladder-retentive systems for treating urothelial carcinoma, with a particular emphasis on recent developments in tumor-targeted intravesical precision medicines.

## 1 Introduction

Urothelial carcinoma is the most common malignancy of the urinary tract and remains a major global health burden, ranking among the ten most frequently diagnosed cancers worldwide in 2022 (1). Approximately 70%–75% of diagnosed cases manifest as non-muscle-invasive cancer (NMIBC), whereas others occur as a muscle-invasive (MIBC) or metastatic disease, carrying a higher risk of dissemination and mortality. For NMIBC, the standard of care is the transurethral resection of the bladder tumor (TURBT) followed by risk-adapted intravesical therapy, most commonly with the immunotherapeutic Bacillus Calmette-Guérin (BCG) and additional chemotherapy. Despite initial therapeutic success, NMIBC is characterized by high 5-year recurrences, as well as treatment challenges such as BCG intolerance or unresponsiveness and chemotherapy resistance, leading to repeated therapy cycles associated with high costs and patient burden. In such cases, especially in high risk disease stage, radical cystectomy is the most effective therapeutic option, a procedure that is however associated with a significant risk of increased morbidity and impact on the patient's quality of life, highlighting the need for treatment alternatives (2, 3). Beyond employing innovative pharmacological modalities, such as combinations with checkpoint inhibitors (4), development of recombinant BCG strains (5, 6) or oncolytic viruses (7–11), improved formulations and drug delivery systems can contribute to more efficient and precise intravesical cancer therapy (5, 12–14).

Catheterization through the urethra enables direct drug administration to the tumor sites, while surpassing systemic distribution and reducing side effects (15). Consequently, intravesical administration allows for application of therapeutics that are unsuitable for systemic use due to associated adverse effects (16). The efficacy of intravesical therapy is influenced by several factors related to the drug, the drug formulation and the urothelial barrier. The urothelium is a highly specialized epithelial tissue characterized by high integrity and low permeability. Superficial umbrella cells are tightly linked, forming the inner lining of the urothelium and preventing paracellular transport of substances. Additionally, the mucus layer rich in negatively charged glycosaminoglycans on the luminal surface contributes to an additional protective permeability barrier (15). Further, continuous urine production and voiding, dilute and eliminate drugs from the bladder rapidly, reducing the residence time and local drug concentration, necessitating repeated dosing (2, 17, 18).

Strategies to overcome these pharmacokinetic challenges include 1. prolonging intravesical drug retention despite voiding 2. promoting drug accumulation at the bladder wall or, more selectively, near the target cancer cells, 3. enhance penetration through the bladder mucosa or into the tumor tissue, and 4. potentially accelerating the onset of pharmacological action by promoting cellular uptake before drug dilution and elimination occur. Intravesical retention strategies can be classified into depot-forming systems with sustained release after bladder instillation, mucoadhesive systems that adhere to extracellular components of the bladder mucosa and actively targeted formulations, that interact with motifs overexpressed by cancer cells (Figure 1). While passive depots enable drug retention to decrease administration frequency, mucoadhesive and targeted formulations additionally enhance local drug concentration and contact time at the urothelial lining. Although both mucoadhesive and active targeted strategies are based on a bioadhesive mechanism, mucoadhesive systems generally rely on wide-spread interaction with mucosal tissue, while active tumor targeting employs highly selective interactions with biochemical entities predominantly expressed by target cancer cells. In the ideal case, active targeting combines prolonged intravesical drug retention with selective local accumulation near the desired target cells and may even accelerate cellular uptake and pharmacological kinetics (15, 18). However, it should be noted that the outermost layer of the bladder mucosa or tumor rim does not necessarily represent the ultimate pharmacological target location: Therapeutic agents generally must further cross the mucus layer, urothelium, or tumor tissue barriers to unleash optimal efficacy. In this regard, strong mucoadhesion or superficial binding to tumors may even restrict further mobility of drugs unless they are released on site and possess sufficient permeability themselves. All the aforementioned systems can be combined with controlled release mechanisms that respond to specific physiological or pathophysiological stimuli, thereby enabling liberation of therapeutic agents. In case of covalently conjugated drugs, the concept of “bioresponsive release” is exemplified by the diverse range of linker chemistries that have been developed. These linkers are designed to undergo cleavage under defined microenvironmental conditions, such as changes in pH or redox potential, or in the presence of characteristic enzymes, thereby triggering the release of conjugated payloads (19, 20). Bladder cancer developing from the urothelial tissue and growing into the lumen, is covered by a mucus layer which, together with the tumor physiological barrier, is a significant permeability hindrance for instilled drugs. Upon bladder cancer progression, formation of tight junctions is often dysregulated or dysfunctional, therefore penetration of drugs into the tumor tissue is mainly governed by paracellular transport, transcytosis or direct transfer via gap junctions or tunneling nanotubes between adjacent cells (21). When a drug molecule has sufficient intrinsic permeability across the mentioned biological barriers, positioning a drug reservoir close to the site of action, where the drug is released locally, can already improve efficacy. However, if the individual active compound is unable to reach its target site in the tumor tissue in sufficient amounts, advanced formulation techniques may be required to enhance tissue penetration (22).

**Figure 1:**
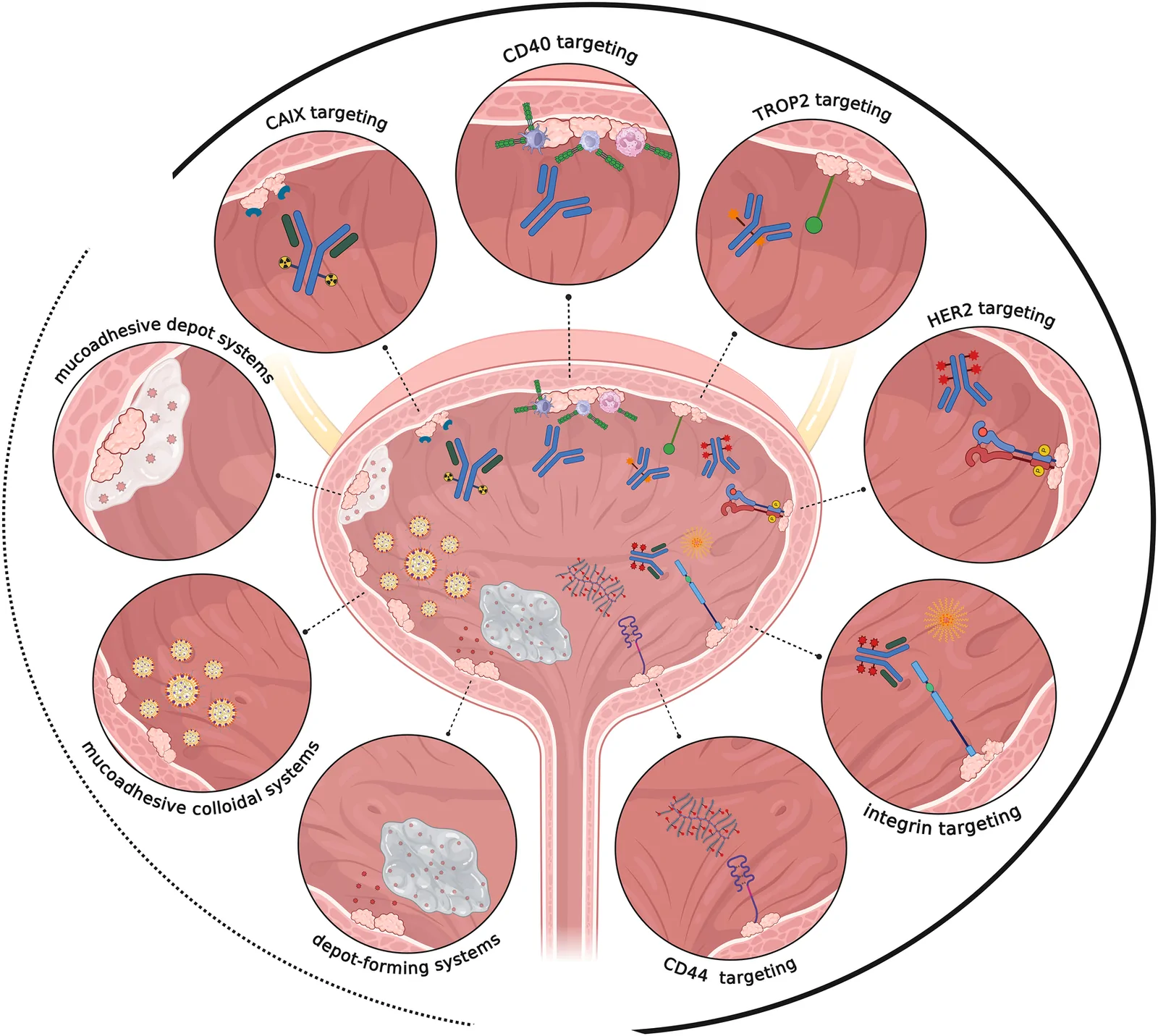
Overview of bladder-retentive systems with a focus on selected actively targeted formulations. Depot-forming systems primarily prolong intravesical drug retention; mucoadhesive formulations enable drug retention and increase drug accumulation at the urothelium; targeted formulations additionally aim for selective deposition at or uptake into cancer cells. Dashed line: non-selective deposition; solid line: selective deposition. [211AT]-giretuximab (CAIX); ADC-1013 & 2141-V11 (CD40); sacituzumab tirumotecan (TROP2); disitamab vedotin (HER2); PLZ-4-paclitaxel micelles & PF-0852667 (integrins); ONCOFID-P-B (CD44).

## 2 Depot and mucoadhesive formulations

### 2.1 Intravesical depot-forming systems

Drugs or drug-containing particles can be incorporated into intravesical hydrogel depot-forming systems to increase bladder residence time and prolong drug retention (23–28) (Figure 1). Generally, hydrogel systems are designed to be biocompatible, non-toxic, highly hydrated like natural tissue, and degradable by enzymes into non-toxic products or dissociate over time (29–31). By controlling the gel composition, crosslinking characteristics and degradation behavior, sustained or triggered drug release kinetics can be achieved to possibly reduce the need for repeated applications (29–31).

Poloxamer is a particularly relevant polymer for *in situ* depot formation, since it can undergo a sol-to-gel transition following administration. At low temperatures, the PEO-PPO-PEO triblock copolymers exist as individual polymer chains in solution. Above the sol-to-gel transition temperature, the PPO blocks become increasingly hydrophobic, promoting micelle formation and assembly into a three-dimensional, gel-forming network (32). The clinical relevance of intravesical hydrogel-based depot formulations is demonstrated by the FDA approval of UGN-102 in June 2025, an intravesical mitomycin reverse-thermal hydrogel formulation (Zusduri, UroGen Pharma) for adult patients with recurrent low-grade intermediate-risk NMIBC (33). The formulation is intravesically administered as a liquid at room temperature, solidifies at body temperature and forms a depot in the bladder, that slowly disintegrates within 6 h, prolonging exposure of the local tumor site to mitomycin (31, 34, 35). The formulation contains poloxamer 407 as the main thermo-gelating component, as well as hydroxypropyl methyl cellulose, polyethylene glycol and water (34, 35).

### 2.2 Intravesical mucoadhesive systems

Intravesical mucoadhesive formulations additionally aim to increase drug retention by promoting contact and adhesion to the urothelial mucosa (Figure 1). Mucoadhesion is a specific form of bioadhesion that describes binding to mucus-covered membranes or mucosal tissues. Physiologically, mucus forms a protective barrier on the apical side of epithelial tissues, with high-molecular weight glycoproteins (mucins) as the main structural components (36). Mucoadhesion can be mediated by several mechanisms, such as non-covalent (electrostatic, H-bonds, hydrophobic) interactions or covalent bonding (36).

Mucoadhesive drug delivery systems are diverse but generally contain natural or synthetic polymers, which either possess intrinsic mucoadhesive properties or serve as a scaffold for modification with adhesive structural motifs. Commonly investigated polymers include cellulose derivatives, such as methylcellulose and carboxymethyl cellulose (23), chitosan (24, 37–41), polypeptides (28), gelatine (42) and synthetic polymers, such as poly(amidoamine) (PAMAM) dendrimers (43) and modified polyethylene glycol (PEG) or poly(lactic-*co*-glycolic acid) (44, 45).

Beyond monolithic depots, colloidal drug delivery systems can also be engineered to exhibit mucoadhesive properties. Carriers, such as microspheres, microparticles and nanoparticles have shown promising results as intravesical delivery vehicles (24, 26, 37–39, 42–46). They aim to combine mucoadhesion with the size-dependent advantages of nano- and microformulations, such as site-specific drug delivery, improved stability and drug solubility (47). Especially particle systems based on chitosan and thiolated chitosan, have been extensively investigated for intravesical use in preclinical settings. These formulations have shown promising *in vitro* and *in vivo* results as carriers with mucoadhesive, permeation enhancing and release controlling properties (24, 26, 37–41, 46). The interaction of the polycation chitosan with the mucosa is complex and involves electrostatic, hydrogen and hydrophobic interactions, as well as solvent, pH, ionic strength and temperature dependence (36). Thiolated chitosan exhibits even stronger mucoadhesive properties, as covalent disulfide bonds are formed with cysteine-rich mucins (48).

Drug delivery can proceed via internalization of the mucoadhesive formulation followed by intracellular release, or via liberation at the surface of the urothelium. Mucoadhesive gels and microparticles primarily reside on the urothelial surface and release the drug adjacent to the urothelial tissue after which tissue penetration is governed by the drug's physicochemical properties. Concurrently, a fraction of payload is also released into the bladder lumen, where dilution and washout limit exposure. In this context, mucoadhesive formulations can even represent an additional diffusion barrier covering the urothelium which impedes re-absorption of dissolved drugs from the cavity (49).

## 3 Intravesical targeted drug delivery

Targeted intravesical drug delivery specifically exploits overexpressed structures on urothelial cells, such as TROP2, HER2, CD44 or integrins, using high affinity ligands like antibodies, peptides or polymers, to specifically deliver chemotherapeutic drugs predominantly to malignant cells while reducing off-target effects on healthy urothelial tissue (Figure 1). The principal advantage of drug targeting is enhanced on-target activity. Achieving meaningful off-target reduction demands rigorous design and manufacturing of targeting ligands possibly combined with tumor-specific release mechanisms. Many putative target moieties are not exclusively expressed on tumor cells but are also present, at lower levels, in healthy tissues, inevitably leading to some off-target effects (50). While high-affinity ligands are often desired for active targeting, very strong interactions can reduce target cell specificity by promoting binding even at low receptor densities in healthy tissues. By engineering moderate-affinity ligands into multivalent architectures, a receptor-density threshold can be created in a way that stable binding occurs preferentially to cancerous tissue (51). Other advanced approaches even go beyond sparing healthy tissue from cytotoxic drug deposition and combine tumor-targeted drug delivery with specific cytoprotection of non-malignant tissues (52). Moreover, levels of target overexpression can vary between patients, tumor subtypes and over the course of tumor progression even within the same patient and individual tumor lesions. Enfortumab vedotin (EV) is an antibody-drug conjugate (ADC) targeting NECTIN4 which is approved for the treatment of urothelial cancer. In MIBC NECTIN4 expression is reported to be heterogenous across molecular tumor subtypes and between primary and metastatic sites. Yu et al. even described spatial differences within single tumor lesions (53). It has to be noted, that this can have potential clinical relevance regarding efficacy of EV (54). Another critical aspect is that enrichment of drug conjugates at the tumor surface may not be sufficient to tackle cancer cells located in deeper tissue layers. For this reason, ligands are being specifically investigated in drug or carrier conjugates that, following tumor-cell selective endocytosis, facilitate deeper tissue penetration via transcytosis or other transfer mechanism between neighboured cancer cells (49). These differential limitations may necessitate careful investigation of the tumor penetration of targeted formulations as well as the determination of individual receptor expression profiles prior to application. Despite the challenges of sufficient selectivity, inter- and intraindividual heterogeneity as well as tumor tissue uptake, active targeting can lead to a therapeutic efficacy that would otherwise require hardly tolerable drug doses (55).

Several ADCs, including trastuzumab deruxtecan (Enhertu®) and enfortumab vedotin (Padcev®) are approved for the systemic therapy of advanced urothelial carcinoma, highlighting the clinical value of an antigen-directed payload delivery (56, 57). Extending these positive clinical outcomes to local bladder cancer therapy, multiple ADCs that target overexpressed motifs, including HER2 and TROP-2, are currently under clinical investigation for intravesical use. Besides tumor-homing, ADCs can additionally modulate signalling pathways by antibody-antigen interaction that initiate tumor cell death. Other approaches use drug-polymer conjugates (e.g., hyaluronic acid targeting CD44), immunomodulatory antibodies or peptide-based nanoformulations that exhibit high affinity to overexpressed motifs on cancerous urothelial cells. By combining intravesical administration with molecular targeting, these strategies aim to minimize systemic exposure and associated adverse effects, while enhancing local delivery to cancerous cells, cellular uptake and potentially additional therapeutic effects.

### 3.1 CD44

CD44 is a non-kinase cell-surface glycoprotein, that mediates cell-cell and cell-matrix interactions, adhesion and migration by binding of ligands such as hyaluronic acid (HA), collagen, osteopontin or fibronectin, thereby modulating signaling pathways such STAT3, mTOR, ERK, NF-*κ*B. Dysregulated CD44 expression has shown to contribute to cancer initiation and progression (58–60). The standard isoform (CD44s) is most prevalent and other isoforms (e.g., CD44v3, CD44v6, and CD44v8-10) are generated by alternative splicing of the variable exons 6–15. These isoforms can exhibit tissue-specific overexpression and adopt new functionalities, which can be associated with poor prognosis, aggressive disease development and metastasis (58, 60). CD44 can also alter the cytoskeleton, promote epithelial to mesenchymal transition (EMT) and enhance drug resistance or apoptosis-inhibition. Taking advantage of CD44 as a receptor for tumor homing, therapeutic agents are conjugated to HA, to potentially enhance receptor-mediated uptake by urothelial cancer cells after intravesical administration (58, 59, 61–64).

To simultaneously treat and visualize bladder cancer tissue in a theranostic fashion, Qiu et al. prepared core-shell nanoparticles, consisting of a doxorubicin-loaded polydopamine core and a shell of HA labeled with the near-infrared (NIR) fluorescent probe IR780 (HA-IR780). An acid/hyaluronidase-responsive release mechanism of the formulation, combined with CD44 binding of HA-IR780 promoted intravesical retention, tumor accumulation and penetration, as well as growth suppression in an orthotopic bladder cancer model in rats (64).

Combining mucoadhesion with CD44-targeting of bladder cancer cells, HA-coated iron oxide nanoparticles (IONPs) embedded in a mucoadhesive PAMAM dendrimer and aldehyde hyaluronic acid (AHA) hydrogel were preclinically investigated. Under NIR irradiation, the delivered IONPs generated heat in a photothermal conversion process desired to induce remodeling of the extracellular matrix and increase tumor tissue penetration (63). Furthermore, delivering iron oxide to cancer cells can induce ferroptosis, an iron-dependent process that, under conditions of iron overload and insufficient antioxidant activity, promotes membrane phospholipid peroxidation and cell death (65). The formulation achieved up to 50-fold higher iron accumulation in the affected area compared to systemic administration (63).

ONCOFID-P-B (Fidia Pharmaceuticals) is a paclitaxel-HA conjugate formed via ester bonds between the carboxyl groups of HA and paclitaxel, improving water solubility of the hydrophobic free drug (61, 66). Additionally, HA moieties bind to CD44 and enhance internalization of paclitaxel (61, 62, 67). ONCOFID-P-B is currently clinically evaluated for the treatment of BCG-unresponsive bladder carcinoma *in situ* via intravesical administration in a phase III trial (NCT05024773) (61).

### 3.2 HER2

Human epidermal growth factor receptor 2 (HER2) is a member of the HER transmembrane receptor tyrosine kinase family. By activation of intracellular signaling pathways such as MAPK or PI3K/Akt, HER2 influences cell proliferation, motility and survival. Particularly in breast and gastroesophageal cancer, HER2 is known as an oncogene, as well as prognostic and predictive marker. Bladder cancer can also be associated with HER2 overexpression, however, disease heterogeneity, ranging from NMIBC to MIBC and metastatic disease, together with non-standardized HER2 assessment and reporting, have yielded inconsistent findings that are difficult to translate into clinical practice (68, 69). A recent systematic review and metanalysis by Guo et al. found that in NMIBC, HER2 overexpression correlates with disease progression, multifocality and more severe tumor grade, whereas in MIBC HER2 expression did not correlate with the tumor grade, suggesting a change with disease progression or masking by other alterations (68). Notably, HER2 expression correlates strongly with lymph node metastasis, supporting a potential role in lymphatic spread and poor prognosis (68, 69).

Disitamab vedotin (RC48) is an ADC with an anti-HER2 monoclonal antibody (mAb) and a protease-cleavable valine-citruline linker bearing the monomethyl auristatin E (MMAE) payload (14). Systemic RC48 showed promising potential for the treatment of urothelial carcinoma and is under evaluation in a phase III study (NCT07207824) (70), as well as in several other studies in combination with other tumor therapeutics (71–73). Leveraging the advantages of intravesical drug administration, Hong et al. evaluated RC48 for intravesical bladder cancer therapy. *In vitro,* RC48 had promising cytotoxic activity in HER2 positive bladder cancer cell lines (e.g., 5637 cells) and in an orthotopic mouse model intravesical application showed superior *in vivo* tumor inhibition and tolerability at therapeutic doses vs. other non-targeted cytotoxic agents (e.g., epirubicin) (74). Based on these preclinical results, a phase I/II clinical study is currently evaluating the safety, efficacy and pharmacokinetics of intravesical RC48 administration in patients with HER2-positive NMIBC (NCT06378242) (75, 76).

### 3.3 TROP2

Trophoblast cell surface antigen 2 (TROP2) is a transmembrane glycoprotein overexpressed across many epithelial malignancies and is associated with tumor growth, proliferation and metastasis. TROP2 influences several oncogenic pathways such as MAPK, PI3K/Akt and IGF-1 and its expression is modulated by transcription factors such as TP63, AIRE and ERG. Particularly TP63 is upregulated in bladder cancer cells, enhancing cell proliferation and adhesion. TROP2 expression is low in non-malignant urothelium, increases in NMIBC and is highest in MIBC, making it a potential target, even in advanced disease stages (77).

Taking advantage of TROP2 tumor homing, MK-2870 (sacituzumab tirumotecan) is an ADC comprising a humanized anti-TROP2 mAb, a pH sensitive sulfonyl pyrimidine-CL2A-carbonate linker and SN-38, the active metabolite of irinotecan, as payload (78, 79). A phase I/II study is currently recruiting to assess the maximum tolerated intravesical dose in patients with intermediate-risk NMIBC (NCT06637423) (80, 81).

### 3.4 CAIX

Carbonic anhydrase IX (CAIX) is a catalytic enzyme, that regulates the pH particularly under hypoxic conditions by catalyzing the hydration of carbon dioxide (CO_2_) to bicarbonate. CAIX has been shown to be upregulated in several cancer types such as breast, pancreatic, lung and bladder cancer and additionally has been identified as a diagnostic urine marker for bladder cancer (82, 83). Tödenhofer et al. aimed to evaluate the potential of CAIX as a therapeutic target in urothelial carcinoma and confirmed that CAIX was significantly elevated in cancerous bladder tissue and low in benign tissue, although their study did not reveal a correlation between the tumor stage and CAIX expression. Furthermore, inhibition of CAIX did not inhibit tumor growth in bladder cancer cells *in vitro* or in an *in vivo* mouse model (82). However, a lack of intrinsic disease modulating properties does not exclude CAIX from being a tumor-selective targeting motif.

A phase I trial is currently planned to assess the safety, tolerability and response of [^211^AT]-Girentuximab (ATO-101™) for intravesical therapy of nonresponsive NMIBC (NCT07260162) (84). Astatine-211 (^211^AT) is an alpha emitter that exhibits high toxicity due to its linear energy transfer but has a short half-life of 7.2 h. The short path of alpha particles, typically less than 100 µm, makes alpha-emitting radionuclides particularly suitable for targeting superficial bladder cancer cells, such as those in NMIBC, while radiation exposure outside the bladder is minimized (85). Combined with the CAIX-targeted antibody girentuximab, the main aim is to locally eradicate BCG unresponsive tumor tissue (86).

### 3.5 CD40

CD40 is a promising target for cancer immunotherapy, as it is a broadly expressed immune-cell stimulatory receptor. It is a member of the tumor necrosis factor (TNF) cell-surface receptor superfamily expressed on B cells, as well as dendritic cells (DCs), neutrophile granulocytes (NCs) and macrophages (87, 88). Binding to its ligand CD40L activates a cascade of innate and adaptive immune pathways – leading to macrophage activation and enhanced proinflammatory cytokine production, T-cell activation, NCs infiltration, DC dependent T-cell activation as well as antibody secretion by B cells – ultimately resulting in cell mediated tumor death.

Promising agonistic anti-CD40 antibodies take advantage of the immune cascade, inducing tumor cell elimination (87). However, systemic administration often results in dose-limiting immune adverse effects such as cytokine release syndrome or thrombocytopenia, due to broad systemic CD40 expression (16, 87, 89). To achieve localized restriction, intratumoral injection or other local administration may be considered, though this is not always possible (87). The anatomy of the urinary bladder allows localized intravesical application, which may potentially increase the therapeutic efficacy while avoiding immune-related systemic toxicity. Furthermore, CD40 has been detected on both urothelial bladder cancer cells and tumor-infiltrating immune cells, making it an attractive therapeutic target (90, 91).

Mangsbo et al. developed ADC-1013, a human agonistic CD40-antibody, for local intravesical administration. *In vitro* experiments revealed a dual mode of action: firstly, inducing direct killing of CD40-positive tumor cells and secondly activation of cytotoxic T-cells, especially via activation of DCs and upregulation of co-stimulatory factors such as CD80 and IL12. *In vivo* experiments using a mouse tumor model showed long-lasting anti-tumor effects and the establishment of immunological memory (16). Another anti-CD40 antibody, 2141-V11, with optimized agonistic CD40 activity, is currently under evaluation in phase I for intravesical administration to patients with BCG unresponsive NMIBC (NCT05126472) (92, 93).

### 3.6 Integrin

Integrins are a family of 24 heterodimeric transmembrane glycoprotein adhesion receptors, formed by variable pairing of 18 *α*-and 8 *β*-subunits, with each heterodimer showing a distinct expression pattern and function. Their extracellular domain mediates ligand-binding, while the cytoplasmic tail enables downstream signaling. Through “inside-out” and “outside-in” signaling pathways, integrins are involved in different processes, including cell differentiation, migration, adhesion, immunity modulation, homeostasis regulation or wound healing. Altered integrin expression has been linked to tumor progression, angiogenesis, migration, invasion and metastasis in several cancers, including breast, lung, liver and gastric malignancies (94–96). Based on its fundamental biological roles, integrins are also being investigated as potential targets for local drug delivery in bladder cancer.

PF-08052667 exemplifies this strategy: it is an ADC with an anti-*β*6 humanized IgG1 mAb linked to the cytotoxic agent MMAE via a PEGylated glucuronide linker. In preclinical intravesical models, a bladder prewash with 0.1% n-dodecyl-*β*-D-maltoside (DDM) enhanced urothelial MMAE delivery and reduced tumor burden, even with shorter dwell times. Although v*β*6 was detected in both healthy and cancerous human bladder samples, the researchers argue, that the normal urothelium is less vulnerable to MMAE because of its lower proliferative activity. The intravesical application may also minimize exposure to rapidly dividing tissues located outside the urinary bladder such as the skin and gastrointestinal mucosa, thereby reducing adverse effects (97). A phase I clinical trial is evaluating the intravesical use of PF-08052667 as a single agent and in combination therapy with a DDM prewash for bladder cancer treatment (NCT07206225) (98).

Another approach uses recombinant noninfectious bacterial minicells VAX-014, that target *α*5*β*1 or *α*3*β*1 integrin heterodimers associated with NMIBC. Minicells are spherical, bacteria-derived nanoparticles, that contain all bacterial components, but lack the bacterial chromosome, making them unable to replicate (99). The integrin-targeted system is engineered to deliver perfringolysin O (PFO), a cholesterol-dependent membrane pore-forming protein, to cancerous cells, leading to cell membrane disruption and lysis (99, 100). A phase I study has been completed to assess the safety, tolerability and activity of VAX-014 for the treatment of NMIBC (NCT03854721), however results have not yet been published (100).

A peptide-based targeting strategy of integrins are paclitaxel-loaded nanoscale micelles (PPM) coated with PLZ-4, a cyclic peptide (cQDGRMGFc) identified by high throughput screening that binds preferentially to *α*V*β*3 expressing bladder tumor cells (101, 102). The micelle building block PLZ-4-PEG5k-CA8 consists of a polyethylene glycol (PEG5k) segment, that is linked to eight cholic acid units (CA8) forming a hydrophobic core of micelles which display the targeting peptide PLZ-4 on the outer surface. Co-formulation with paclitaxel in aqueous solution drives self-assembly and encapsulation of the API within the formed micelles (101). Two phase I clinical studies are currently recruiting to determine the safety, effectiveness and dose (NCT05519241 and NCT06173349) (103, 104).

## 4 Discussion

Intravesical drug delivery is an attractive option for treating urothelial malignancies, as it achieves high local concentrations at the disease site while minimizing systemic exposure and associated toxicity. Mucoadhesive formulations are of particular interest due to their ability to resist urinary washout thereby extending dwell time and increasing urothelial drug deposition. The FDA's approval in 2025 of UGN-102, a reverse-thermal hydrogel depot formulation providing sustained mitomycin-release, highlights the considerable clinical potential of the concept (33). Additionally, targeted delivery platforms are emerging as new precision medicines which can concentrate pharmacologic activity within cancerous tissues and may accelerate the onset of action by employing receptor-mediated endocytosis mechanisms. A variety of targeted intravesical formulations have reached the stage of clinical evaluation. These include ADCs directed against HER2 (75), TROP2 (80), CAIX (86) and integrin *β*6 (98); the CD44-targeting paclitaxel-hyaluronic acid conjugate ONCOFID-P-B (61); integrin-targeted platforms such as VAX-014 (100) and PLZ-4 coated paclitaxel micelles (103, 104); and the immunomodulatory anti-CD40 antibody 2141-V11 (92). Preclinical studies have also investigated the potential of HA-functionalized nanoformulations to enable CD44-mediated tumor accumulation (63, 64). To facilitate the clinical translation of targeted drug delivery systems, particular attention should be given to interindividual heterogeneity and patient stratification to identify those most likely benefiting from targeting strategies, as well as comparative evaluation against established treatment options. The local intravesical route can make agents clinically usable that would otherwise be precluded by dose limiting adverse effects in systemic administration. Consequently, a further opportunity for advancing intravesical treatment of bladder cancer is the repurposing of drugs or drug conjugates originally investigated for systemic use. When reformulated for local administration, many of these liabilities may be mitigated, potentially unlocking clinically meaningful activity in the bladder. As outlined in the article, owing to the characteristics of the bladder and intravesical administration route as well as pathophysiology of bladder cancer, an optimal delivery system has to meet a wide range of desired requirements, such as drug retention, drug accumulation in proximity to the site of action, tissue penetration and controlled drug release. Modern chemical engineering theoretically provides the necessary toolkit for designing specifically adapted, multifunctional drug delivery systems. However, the clinical translation of complex drug formulations is associated with additional practical challenges since conventional procedures developed for well-defined small molecule formulations do not translate directly. As complexity increases, GMP compliant, reproducible manufacturing, feasible scale-up, ensuring reliable quality control, storage stability, and application in routine clinical settings become substantially more sophisticated. Nonetheless, complexity *per se* does not necessarily prevent clinical use. Instead, efforts and benefits must be carefully balanced, always with the well-being of patients remaining the primary focus. For indications with a clear benefit or limited therapeutic alternatives, even in small patient cohorts, sophisticated innovative formulations can progress to clinical translation as demonstrated by ADCs with triggered drug release, nanomedicines, genome-editing platforms, and cell therapies that are currently in clinical trials or already approved. As a concluding framework, key considerations and critical design parameters for the development of future intravesical therapies discussed throughout the article are summarized: intravesical formulations for the treatment of bladder cancer are desired to enable (i) drug retention, (ii) prevention of urine-mediated dilution or washout, (iii) tumor-selective accumulation, (iv) drug penetration to the target sites, (v) minimized exposure or active protection of healthy urothelium, (vi) manufacturability, and (vii) applicability in routine clinical settings. By providing an overview of the evolution of intravesical formulations - from passive drug depots and mucoadhesive systems to actively targeted delivery platforms - the aim of this article is to underscore their potential to advance bladder cancer therapy.
